# Rocking the Boat: Damage to Eelgrass by Swinging Boat Moorings

**DOI:** 10.3389/fpls.2017.01309

**Published:** 2017-07-25

**Authors:** Richard K. F. Unsworth, Beth Williams, Benjamin L. Jones, Leanne C. Cullen-Unsworth

**Affiliations:** ^1^Seagrass Ecosystem Research Group, College of Science, Swansea University Swansea, United Kingdom; ^2^Project Seagrass Cardiff, United Kingdom; ^3^Sustainable Places Research Institute, Cardiff University Cardiff, United Kingdom

**Keywords:** eelgrass beds, boating, disturbance ecology, seagrass ecology, conservation biology

## Abstract

Seagrass meadows commonly reside in shallow sheltered embayments typical of the locations that provide an attractive option for mooring boats. Given the potential for boat moorings to result in disturbance to the seabed due to repeated physical impact, these moorings may present a significant threat to seagrass meadows. The seagrass *Zostera marina* (known as eelgrass) is extensive across the northern hemisphere, forming critical fisheries habitat and creating efficient long-term stores of carbon in sediments. Although boat moorings have been documented to impact seagrasses, studies to date have been conducted on the slow growing *Posidonia* species’ rather than the fast growing and rapidly reproducing *Z. marina* that may have a higher capacity to resist and recover from repeated disturbance. In the present study we examine swinging chain boat moorings in seagrass meadows across a range of sites in the United Kingdom to determine whether such moorings have a negative impact on the seagrass *Zostera marina* at the local and meadow scale. We provide conclusive evidence from multiple sites that *Z. marina* is damaged by swinging chain moorings leading to a loss of at least 6 ha of United Kingdom seagrass. Each swinging chain mooring was found to result in the loss of 122 m^2^ of seagrass. Loss is restricted to the area surrounding the mooring and the impact does not appear to translate to a meadow scale. This loss of United Kingdom seagrass from boat moorings is small but significant at a local scale. This is because it fragments existing meadows and ultimately reduces their resilience to other stressors. Boat moorings are prevalent in seagrass globally and it is likely this impairs their ecosystem functioning. Given the extensive ecosystem service value of seagrasses in terms of factors such as carbon storage and fish habitat such loss is of cause for concern. This indicates the need for the widespread use of seagrass friendly mooring systems in and around seagrass.

## Introduction

Seagrass meadows commonly reside in shallow sheltered embayments, typical of the locations that are attractive for mooring boats. These boat moorings present a source of small scale long-term repeated physical disturbance to seagrass (Hastings et al., 1995; Macreadie et al., 2015). Seagrasses take root in soft sediments where the heavy chains associated with moorings can uproot rhizomatous tissue and tear shoots (Bourque et al., 2015). Seagrasses need sheltered conditions which reflects the susceptibility of their anchoring within this environment to physical disturbance (Hemminga and Duarte, 2000).

Disturbance occurs as the mooring chain rotates around a central anchor point with tidal and wind induced movements associated with the buoy. This movement is typically exacerbated by the attachment of a boat which acts as a sail. The movement then causes the chain to drag along the substrate and can lead to declines in seagrass (Milazzo et al., 2004). Damage to the seagrass leaves, or sediment resuspension resulting in reduced light levels in the water column can lead to decreased photosynthesis levels (Di Carlo and Kenworthy, 2008). Along with reduced light levels through increased sedimentation in the water column, the direct physical impacts of moorings can cause long-term losses of other resources from the sediment such as organic carbon (Serrano et al., 2016). Physical disturbance can also prevent or limit the recovery of a damaged meadow, for example, *Zostera marina* seedlings exposed to physical disturbance encountered a threefold higher mortality rate than seedlings protected from disturbance (Valdemarsen et al., 2010).

The life history traits of seagrasses changes between species and across the four families (Unsworth et al., 2015). This leads to key differences in the mechanical strength of seagrass species and how they might be affected by physical stress (de los Santos et al., 2016). Differences in life history traits may ultimately lead to dissimilarities in the ability of different species to remain resilient (resist and recover) to the impacts of disturbance from a standard chain mooring. Due to the global use of boat moorings their damage to seagrass meadows has been quantified in a variety of locations, but the majority of these are within *Posidonia* spp. meadows, the slowest growing of the seagrasses. Boat moorings placed in *Posidonia oceanica* meadows have been found to result in a decline in shoot density and meadow cover and to cause rhizomes to become exposed (Francour et al., 1999; Milazzo et al., 2004; Montefalcone et al., 2008). A range of other studies have also documented the impact of moorings on *Posidonia* spp. (Walker et al., 1989; Hastings et al., 1995; Demers et al., 2013).

*Posidonia* species’ are generally considered to be long lived and slow growers, and therefore slow to recover from stress. Conversely, *Z. marina* is a fast growing species, and rapid recovery from small scale disturbance has been reported (Qin et al., 2016). However, very slow long-term recovery from disturbances such as dredging have also been reported (Hilary et al., 2005). *Z. marina* has a circumglobal distribution throughout the northern hemisphere where it plays a critical role in supporting, amongst others things, fisheries production, carbon storage and sediment stabilization (Jackson et al., 2001; Bertelli and Unsworth, 2014; Mtwana Nordlund et al., 2016). Unfortunately there is extensive and growing evidence of the large scale loss and degradation of *Z. marina* meadows across its range (Waycott et al., 2009; Jones and Unsworth, 2016; Lefcheck et al., 2017). Understanding how *Z. marina* responds to physical disturbances such as those resulting from boat moorings remains a key issue for its management.

Given that *Z. marina* is relatively tolerant to increased sedimentation when compared to *Posidonia* species’, and coupled with the ability of *Z. marina* to produce large quantities of seeds and exhibit rapid rhizome growth (Erftemeijer and Lewis, 2006) there exists potential for this species to undergo some level of recovery after physical disturbance. *Z. marina* may remain unaffected or impacted to a lesser degree (e.g., have reduced cover rather than complete loss) than other species of seagrass. This is the first study we are aware of that attempts to quantify the impact of boat moorings on *Z. marina* and we argue that given the propensity for moorings to be placed within seagrass habitat and the widespread distribution of this particular species around the globe such information is needed for better informed habitat management.

The present study was conducted in the United Kingdom where understanding threats to the seagrass system at local levels is also of importance. Historical loss of seagrass in the United Kingdom is thought to have been extensive (Wilding et al., 2009) and recent studies have indicated that current populations are in a perilous state (Jones and Unsworth, 2016) and poorly managed (Jackson et al., 2016). To date there exists limited understanding of the key threats to United Kingdom seagrass, specifically the impacts of boat moorings on its abundance.

In this study, we tested the following null hypothesis: *Z. marina* is not impacted at the small (patch) or meadow scales by the presence of boat moorings. The hypothesis was tested by examining seagrass surrounding boat moorings in seagrass meadows from a range of sites in the United Kingdom using a combination of field based assessments and remote sensing techniques.

## Materials and Methods

### Study Sites

**MAP 1:**
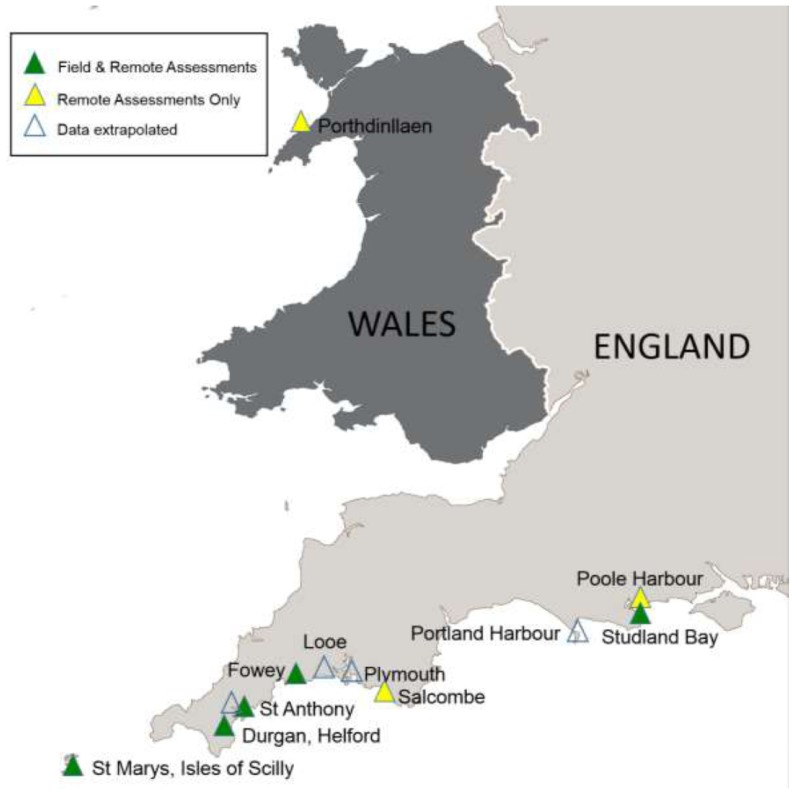
Locations of five sites assessed (*in situ*) to examine the impact (small scale) of swinging boat moorings (green triangles) on seagrass. Two of these five sites were additionally examined to consider meadow scale impacts (Studland Bay and Durgan). Included on the map are three additional sites (yellow triangles) assessed remotely using satellite imagery (Poole Harbor, Porthdinllaen, and Salcombe). The location of four further sites in the United Kingdom (Looe, Plymouth Harbor, Flushing, and Portland Harbor) known to have seagrass containing moorings scars are also shown but unassessed in the present study (open triangles). A further site known to contain these scars is Strangford Lough (not shown).

Detailed field studies on the seagrass surrounding swinging boat moorings were conducted at five study sites located along the South Coast of England (**Map 1**). The five field sites were Studland Bay (50°09′14N, 1°56′20W), Durgan Bay, Helford River (50°06′10N, 5°06′55W), St. Anthony in Roseland, St. Mawes (50°09′14N, 5°00′41W), St. Marys, Scilly (49°55′00N, 6°18′49W) and Fowey (Polruan, River Fowey) (50°19′47N, 6°18′49W) (see **Map 1**). At two of these sites (Durgan and Studland) additional meadow scale data was collected on seagrass status at increasing distances from areas containing boat moorings. Remote studies were conducted at an additional three United Kingdom sites known to have extensive *Z. marina* meadows (Poole Bay, Salcombe, and Porthdinllaen).

### Mooring Assessments

Moorings examined during detailed field studies were all of the swinging type based around one heavy chain riser rotating on a swivel around a concrete or weighted mooring point. The length of the chain and type of chain used appeared to vary widely with no consistent design feature.

**FIGURE 1:**
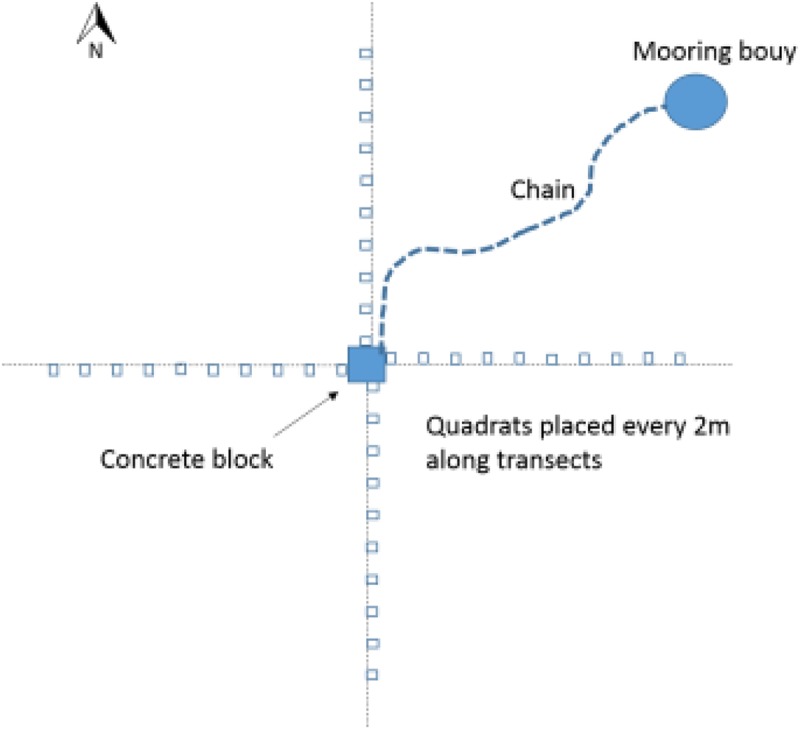
Sampling design used to examine seagrass surrounding each mooring.

Seagrass meadows were sampled using video analysis around selected moorings and in control areas. Control areas were in the same meadow and close to the moorings but not within their immediate influence (e.g., a minimum of 20 m from a mooring). To sample the seagrass a camera frame containing a GoPro Hero 4 mounted above a 0.25m^2^ quadrat was lowered by hand into the seagrass (at low tide) every 2 m along 20 m transects rotated around a central point (See **Figure 1**). This central point was where the mooring was fixed to the seabed and therefore the chain swung around this point. The transect was measured away from each mooring four times, in the N, S, E, and W cardinal directions. Fifteen control seagrass quadrats were also assessed using the frame. Controls were placed at each site in the gaps between moorings at locations beyond the extent of any of the mooring chains where seagrass was continuous. The position of the mooring, whether it was a chain or a rope mooring, and the thickness of the chain was recorded for all the moorings analyzed. Due to the weight of the chains and the presence of boats it was not possible to determine a straight line length of each mooring chain.

### Meadow Scale Assessments

To study the meadow scale impact of moorings, at two sites (Durgan and Studland) the seagrass meadows were sampled over a larger scale. Sampling was conducted within the moorings (control), in line with the sites’ outer mooring (edge or 0 m), and then 50, 100, and 150 m away from the outer mooring in one direction, the sampled areas were kept at a relatively consistent depth. Distances were either measured along the beach or seagrass meadow itself where possible, or by using Google Earth. Coordinates were chosen from the satellite images from Google Earth, at each sampling distance. At each distance 15 quadrats of seagrass were assessed, this was done by hap-hazardly dropping down the camera frame onto the seagrass meadow at points at least 2 m apart.

### Video Analysis

During the analysis of the videos the seagrass cover, seagrass canopy height, and algal cover were all recorded, and these were quantified within the camera frame. This followed the quadrat level methods of SeagrassWatch (McKenzie et al., 2004). Pictorial reference guides were used as standards for the percentage cover measurements for both the seagrass and algae (McKenzie et al., 2004). These guides were used throughout to increase the consistency and accuracy of the results obtained from the video analysis. The height of the canopy was measured using height markers on the camera frame. These markers were placed at 20, 30, 40, 50, and 60 cm. When the tallest seagrass reached beyond the highest marker the canopy height was recorded as 60 cm+.

**FIGURE 2:**
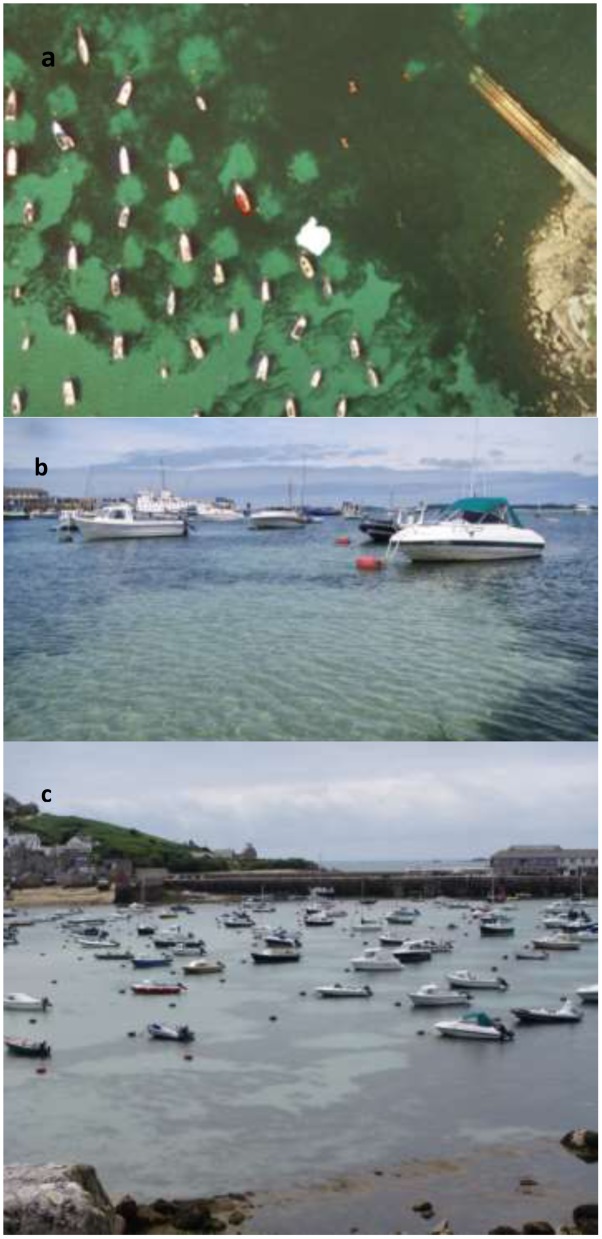
**(a)** Satellite image from Google Earth (2016) of St. Marys, Scilly demonstrating the use of the polygon toll to calculate the size of the scar. **(b)** Image taken of a mooring at St. Marys, Scilly highlighting the distinction between the scarred area and the higher percentage of seagrass cover. **(c)** Image taken from a high view point of the moorings studied in St. Marys, Scilly, due to the close proximity of the moorings measurements up to 20 m often fell into the area of another scar.

The total area surrounding a mooring that was scared of seagrass was calculated. This was defined as the area between the center of the mooring and the substratum where the seagrass cover was less than 10%. Due to sampling taking place every 2 m, the distance used to calculate the scar was between the points where the seagrass cover was less than 10% and where it increased to or above 10%. This scarred distance (measured in meters) was calculated for each direction of the moorings, and the equation 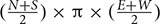 (where N, S, E, and W are the scarring distances in each direction of the compass, North, South, East, and West) was used to calculate the scarred area. This resulted in the creation of a circular to elliptical shaped area (see example aerial image in **Figure 2**). In some cases a cut-off point could not be defined, this was due to the seagrass cover not reaching 10% in the 20 m sample area. The reasons for this could be due to the scarred area exceeding 20 m, or due to the cover of the seagrass bed being low in this area regardless of the mooring. In these cases if a cut-off point could not be determined the sample was removed from the analysis, so as to not overestimate the damage. At some sites, the area of scarring was also calculated using a cut-off point of less than 5% seagrass cover. This was done at St. Marys, Scilly and Fowey and used in conjunction to the remote sensing data.

### Remote Sensing of Moorings

At two of the sites (St. Marys Scilly and Fowey) the scars assessed were additionally quantified using imagery in Google Earth Pro^TM^. This allowed for comparisons between field based and remote based methods to be made. These sites were chosen due to the quality of the satellite images in which the sea state and water clarity allowed for simple assessment of the seagrass (**Figure 2**). The polygon tool on Google Earth was used to calculate the scarred area from the satellite images. The location of the polygon was determined on an object style classification where a clear change in color could be determined (visually). Areas calculated using Google Earth imagery can only be described as broad estimates and are likely influenced by a range of factors including age of photo, season of photo (influencing seagrass cover), and clarity of image.

Moorings where the cut-off point for a direction could not be determined, were not included in the comparison with the field results. This was the case for the third mooring at Fowey. The use of the polygon tool was then extended to calculate the total area of lost seagrass at St. Marys, Scilly, Studland Bay and Fowey, by measuring all of the mooring scars at these sites.

Based on the comparisons between field and remote methods the use of remote assessment was extended to three further sites (where no field based work was conducted), these were Poole Bay, Salcombe, and Porthdinllaen.

### Statistical Analysis

In order to examine small scale effects of moorings on seagrass we used a two-factor analyses of covariance (ANCOVA) to compare the cover of seagrass, canopy height and algae (each metric in a separate analysis) with respect to distance from boat mooring points at different sites. All data were tested for heteroscedasticity and normality using the Shapiro and Bartlett tests. Statistical analysis was carried out using the statistical program Minitab v13 and transformed (arcsine) when necessary. A Pearson’s correlation test was used to test the association between distance away from the mooring and seagrass cover (%), seagrass height, and algal cover (%).

To test for an association between different descriptors of the seagrass meadow at small and meadow scales [seagrass cover (%), algal cover (%), and seagrass height] correlation tests were used. A Pearson’s correlation was used for seagrass cover (%) and seagrass height, and a Spearman’s rank test was used for seagrass cover (%) and algal cover (%).

To investigate the meadow scale impact two-way ANOVA was used to compare both the seagrass cover (%) and algal cover (%) over the different categories [within moorings/control, edge (0 m), 50 m, 100 m, and 150m]. A Tukey HSD test was then used to investigate the individual differences between distances. Linear regression was used to examine the relationship between different methods for assessing the size of the mooring scars in seagrass.

## Results

### Seagrass Condition

**FIGURE 3:**
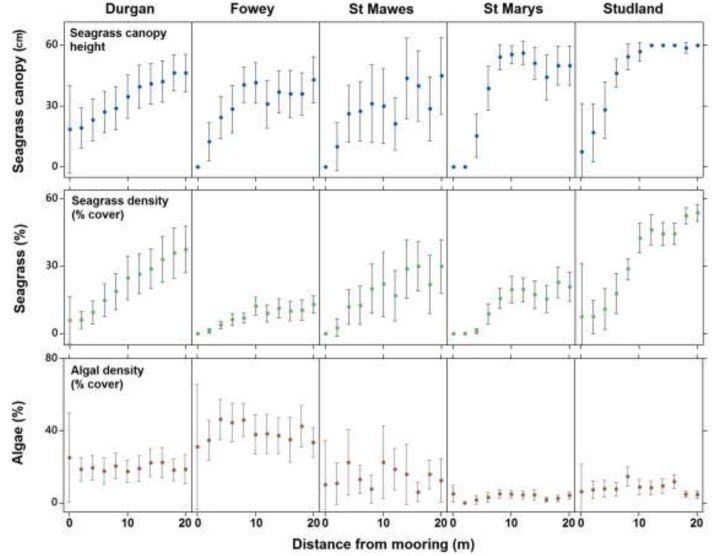
The mean (±SD) seagrass cover, canopy height (cm) and algal cover at each of the five field sites on the south coast of England (St. Marys, Durgan, Studland, Fowey, and St. Mawes) over varying distances from the chain mooring center. Parameters were measured every 2 m from the center of the mooring (0 m) to 20 m.

Seventy nine percent of quadrats observed at the center of chain moorings (0 m) were found to contain no seagrass compared to 15% of samples taken at 20 m away from the mooring and 0% of the samples taken as controls. Average seagrass cover at 0 m was 2.9 ± 8.4%. This increased to 6.7 ± 11.1% cover by 4 m and had reached 17.3 ± 15.1% cover by 8 m away from the mooring. At 20 m away from the moorings seagrass cover was on average 30.3 ± 22.1%. The increasing percentage cover of seagrass along a linear gradient from the center of the mooring at all sites was significant (ANCOVA, *R*^2^ = 0.42, *F*_3,983_ = 80.1, *P* < 0.001, **Figure 3**). No significant differences in this relationship were observed between sites (*p* = 0.68).

Seagrass canopy height also increased significantly with increasing distance from the center of the mooring at all sites (ANCOVA, *R*^2^ = 0.24, *F*_3,983_ = 45.9, *P* < 0.001, **Figure 3**). Again no significant differences in this relationship were observed between sites (*p* = 0.30). Seagrass canopy height at 0 m from the central mooing point was 6.4 ± 15.2 cm, increasing to 44.6 ± 21.2 cm at 10 m from the moorings.

Algal cover increased significantly with increasing distance from the center of the mooring (ANCOVA, *R*^2^ = 0.06, *F*_3,983_ = 3.1, *P* < 0.001, **Figure 3**) but this trend was not consistent as there was a significant effect of site. At Fowey algal cover was 3.9 ± 0.22% within the 20 m radius around the moorings whereas at Studland Bay this was 0.08 ± 0.08% (**Figure 3**).

### Meadow Scale Impact

**FIGURE 4:**
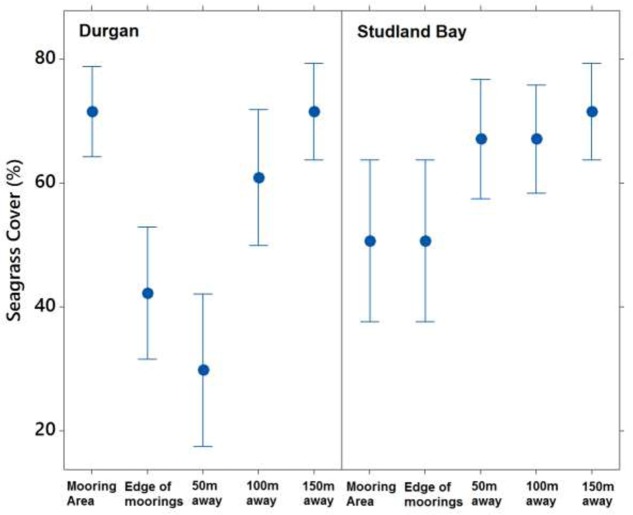
Mean ±SD% seagrass cover at different distances (m) away from the site of mooring at two sites on the south coast of England (Durgan and Studland Bay).

Seagrass cover varied at different distances from the moored area but with no clear pattern (**Figure 4**). At Durgan these differences were significant (*F*_4,70_ = 13.74, *p* < 0.001). A *post hoc* Tukey test at Durgan showed a mixture of differences between distances (**Table 2**).

Seagrass cover also varied significantly with distances from the moored area at Studland Bay (*F*_4,70_ = 3.294, *p* < 0.05). *Post hoc* Tukey test showed that seagrass cover was significantly higher at 50 m than at the control site which was within the mooring area. For all other comparisons there were no significant differences.

### Mooring Scars

The average mooring scar radius (across all sites) was 5.4 ± 3.5 m. This is based upon classifying a scar as an area containing <10% of seagrass cover (**Table 1**). Mean scar radius differed between sites as follows: Durgan 4.7 m (±5.2); at St. Anthony, St. Mawes 3.6 m (±3.4); St. Marys, Scilly 6.75 m (±2.2); Fowey 6 m (±2.2); and Studland Bay 4.25 m (±2.8). Therefore St. Marys, Scilly had the mean largest mooring scars with <10% seagrass cover, while St. Anthony, St. Mawes had the smallest.

**Table 1:** The mean scarred area (m^2^) of the moorings sampled, calculated for each site, this area was defined as the area between the center of the mooring and where the seagrass reached ≥ 10%.

Site	Number of scars assessed	Mean scarred area (m^2^) (±SD)
Durgan	6	167.55 (±229.4)
St. Marys, Scilly	6	147.13 (±73.61)
St. Anthony, St. Mawes	2	39.27 (±37.76)
Studland Bay	4	75.4 (±55.49)
Fowey	5	106.81 (±39.55)
All		121.84 (±125.57)

*Moorings where the seagrass did not reach ≥ 10% in the 20 m radius around the mooring were removed from the analysis < 5%. The mean scarred area (m^*2*^) was also calculated for each site*.

**Table 2:** ANOVA model testing % seagrass cover as a function of distance away from the moored area.

	Difference	Lower	Upper	*p*-value
0 – Control	14	0.13	27.87	0.05
50 – Control	22	8.13	35.85	<0.001
100 – Control	-9.33	-23.20	4.53	0.33
150 – Control	22	8.14	35.86	<0.001
50 – 0	8	-5.56	21.86	0.50
100 – 0	-23.33	-37.20	-9.47	<0.001
150 – 0	8	-5.86	21.86	0.50
100 – 50	-31.33	-45.19	-17.47	<0.001
150 – 50	0	-13.86	13.86	1
150 – 100	31.33	17.47	45.20	<0.001

*The “Control” distance included samples taken from within the mooring area, and 0 m was samples taken from the edge of the mooring area. Seagrass cover was normally distributed and of homogeneous variance. Significant differences are yielded when *p* < 0.05*.

### Scar Calculations: Remote vs. Field Results

**FIGURE 5:**
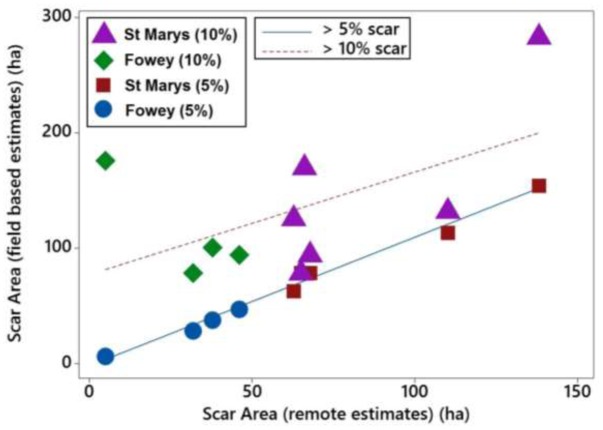
Linear relationships of boat mooring scars at St. Marys, Scilly and Fowey assessed with two different field methods (5 and 10% cover) with scar sizes estimated using remote methods (Google Earth).

The scarred area around the moorings at St. Marys, Scilly and Fowey was calculated using three different methods, seagrass cover < 5% (field based), seagrass cover < 10%, and object based remote methods. Scars observed at 5% seagrass coverage have a strong and significant relationship with those observed remotely (*P* < 0.001, *R*^2^ = 0.98) whereas the relationship is not significant with respect to scars determined at 10% coverage (**Figure 5**). The 5% relationship is close to 1:1 and on average the difference between the predicted (remote method) and the 5% scar is 7.2 ± 10.4 m^2^ (i.e., remote methods underestimating scar size by 7%). Remote methods were unable to accurately predict the scar size at 10% seagrass cover with remote values on average 70 ± 51 m^2^ lower than reflected in the field data.

### Total United Kingdom Seagrass Loss

Based upon the results of this study (the validity at 5% cover for the scar area) further analysis of mooring scars using Google Earth imagery was conducted at Salcombe (Devon), Porthdinllaen (North Wales), and Poole Harbor (Dorset). At each site all moorings that could be measured using remote assessments were quantified. This was done in order to determine a site average for the area of the scar size. Additionally the total number of moorings observed remotely (but not necessarily quantified in area) known to be in an area of seagrass were determined. This information enabled a broad estimate of the total loss of seagrass at each site to be determined and then a minimal estimate for total loss across the eight sites to be calculated. Estimated seagrass loss varied from 409 m^2^ at Salcombe to over 1 ha at St. Marys in the Isles of Scilly (**Table 3**). Mean site scale loss was 4637 ± 3951 m^2^. The high standard deviation indicates the large variability between sites.

**Table 3:** Mean (±SD) of the size of boat mooring scars in seagrass at eight sites around the United Kingdom and the extrapolation of this information to the whole meadow based upon a count of the number of moorings present in seagrass.

	Number of scars assessed	Mean scar area (m^2^)	StdDev of area (m^2^)	Total number of scars	Estimated damage to seagrass (m^2^)
Fowey	21	41.7	20.7	21	876.4
Poole harbor	10	139.9	193.9	70	9790.2
Porthdinllaen	9	114.1	52.2	48	5478.4
Salcombe	6	19.5	5.4	21	409.5
St. Marys, Scilly	68	73.7	70.7	142	10458.3
St. Mawes	5	318.8	252.8	8	2550.4
Studland Bay	13	75.2	48.5	20	1504.769
Helford		167.6	229.4	36	6033.6
**Total (m^2^)**					37101
**Total (ha)**					**3.71**

*Scar areas were determined using Google Earth based on initial validation of method (see **Figure 5**)*.

By applying the mean site scale seagrass loss to five additional sites known to contain permanent moorings in seagrass (Portland Harbor, Flushing, Plymouth, Strangford Lough, and Looe) we have been able to develop a broad United Kingdom wide scale estimate of seagrass loss as a result of standard permanent swinging boat moorings. We suggest that seagrass loss from boat moorings maybe over 3.7 ha for the sites we can assess in the field and remotely (**Table 3**). At the United Kingdom scale we estimate total seagrass loss due to boat moorings to be at least 6 ha. This impact (6 ha) is spread across 13 known seagrass sites containing boat moorings. The likelihood of additional scars, in other sites not included in our study means that United Kingdom wide seagrass loss attributable to swinging boat moorings could be in excess of 6 ha.

## Discussion

This study demonstrates the significant impact swinging chain moorings have on the globally important seagrass species *Z. marina*. We provide conclusive evidence that across multiple sites, *Z. marina* is damaged by swinging chain moorings leading to a minimal loss of at least 6 ha of United Kingdom seagrass and the associated fragmentation of at least nine significant meadows. Given the wide scale historical loss of United Kingdom seagrass, its current perilous state and the limited conservation attention it receives (Jackson et al., 2016; Jones and Unsworth, 2016) our findings add to evidence of an already degraded ecologically vital resource. This loss of United Kingdom seagrass from boat moorings is small but significant at a local scale. This is because it fragments existing meadows and ultimately reduces their resilience to other stressors such as eutrophication (Unsworth et al., 2015; Maxwell et al., 2016).

Each swinging chain mooring was found to result in the loss of 122 m^2^ of seagrass. This loss has the capacity to result in a reduction of the extensive ecosystem service value of seagrasses including carbon storage, invertebrate biodiversity, and fish habitat (Frost et al., 1999; Lilley and Unsworth, 2014; Macreadie et al., 2015).

Our study found that seagrass cover (%) and canopy height increases with increasing distance away from the mooring. Seagrass was mostly present within the area defined as containing a scar, although it was very low in density, with canopy height reduced. The average distance from a mooring that was scarred was 5.4 m. This radial scar of 5.4 m is lower than those recorded for *Posidonia australis* (e.g., 9 m) (Demers et al., 2013). This smaller scarred area may reflect the greater capacity of *Z. marina* to recover relative to the more climax species *P. australis*, alternatively it could also be the result of differences in tidal regimes.

Although the vast majority of chain moorings caused a loss of seagrass (79%), there were some that did not. The reasons for this are not clear, but may be a result of differences in chain thickness and length, duration the mooring had been in place, tidal movement, the amount of use of the mooring, or the size of the boat using the mooring. Improved understanding of these factors may help lead to solutions that can reduce the impact of swinging chain moorings on *Z. marina*.

Although seagrass was recorded within the scarred areas the cover was always less than 5%. This low cover is significant as although it offers little in the way of ecosystem service provision such as fish habitat (McCloskey and Unsworth, 2015) it suggests that recovery might be possible if a mooring is removed or replaced with an ecologically sensitive design (Demers et al., 2013).

The present study did not determine whether low cover was the result of new propagules or seeds, or whether it remained from rhizomes present in the sediment. Such recovery potential requires further consideration. The capacity of the seagrass to recover will depend upon the multiple attributes of the resilience of the system (Unsworth et al., 2015). Some of the sites examined in the present study are considered ecologically largely healthy (e.g., good water quality) except for the mooring and other boat damage (Jones and Unsworth, 2016), meaning that if the moorings were replaced with environmentally sensitive gear there is considerable potential for seagrass recovery.

The results presented here add to the argument regarding the effect moorings have on seagrass in general, supporting (Walker et al., 1989; Hastings et al., 1995; Montefalcone et al., 2008; Demers et al., 2013; Serrano et al., 2016) who report negative impacts of moorings for a variety of seagrass species (see **Appendix 1**).

Although we determined scar area to have a mean diameter of 5.4 ± 3.5 m our study documents how seagrass cover at a distance of between 16 and 20m from the mooring anchor point was lower than that at the control sites. This could indicate the impacted area is larger than just the scarred area, beyond scar damage would be due to the moorings causing seagrass thinning through lower levels of disturbance at the extreme ends of the mooring chain and at distances away from the chain.

Thinning has the potential to impact the ecosystem services provided by seagrass. Studies in Australia have illustrated how long-term carbon stores can be lost as a result of mooring damage (Macreadie et al., 2015; Serrano et al., 2016) and research in Canada has demonstrated invertebrate diversity and abundance is lower in areas with moorings and high levels of recreational boat use (Leatherbarrow, 2006).

Our study found a negative correlation between seagrass cover and algal cover, although this correlation was weak, it suggests that algae may colonize and grow in areas where seagrass has been removed or has faced cover declines through thinning. The presence of macro-algae can limit the recovery potential of seagrass and cause further declines through competition for light and space (Orth et al., 2006). Other ecosystem stressors such as eutrophication can exacerbate this competition by supporting increased algal cover (Short et al., 2006). The influence of light reduction due to shading of the seagrass by the presence of a boat on the moorings may also contribute to such stress.

Seagrass meadows examined in the present study were not found to be impacted at the whole meadow scale by moorings. All impacts were found to be within an area of approximately 20 m radius from the mooring. At the two sites examined for such impacts, seagrass was variable and did not provide a clear consistent pattern of change with respect to distance from the mooring. Seagrass cover at Studland Bay was much more consistent than at Durgan, this may be due to areas of Durgan being anchored on throughout the bed, leading to exacerbated habitat fragmentation (Francour et al., 1999; Collins et al., 2010). The distance from the moored area was also found not to impact the algal cover (%), this again could be due to the quality of the seagrass meadow as a whole not being compromised by moorings.

Satellite imagery and aerial photographs are effective means of assessing seagrass loss (Hastings et al., 1995; Ward et al., 2003). By using a simplistic method of assessing seagrass extent the present study was broadly able to calculate the area of loss of seagrass surrounding a boat mooring (subject to clear imagery). Areas of seagrass scarring determined using remote imagery were not significantly different to those determined in the field (based on seagrass < 5% cover), although some site specific differences were recorded (underestimations with remote methods). We suggest that the depth of the meadow may have influenced this, as has been found in previous studies (Ferwerda et al., 2007). Although the use of such imagery is a quick and cost effective way to monitor the loss of seagrass and has allowed us to propose that at least 6 ha of seagrass in the United Kingdom has been lost due to boat moorings, caution should be taken as this figure is likely an underestimations of seagrass loss.

## Conclusion

We find strong evidence that swinging chain moorings result in a significant loss of *Z. marina*. This loss and impact is isolated to the surrounding area to the mooring and does not translate to a meadow scale. However, an abundance of moorings, as is seen throughout the United Kingdom and globally at sites containing seagrass, can lead to a significant loss of habitat and ultimately ecosystem functioning. In spite of the higher growth and recovery potential of *Z. marina* relative to species of *Posidonia* spp. impacts are still of the same magnitude, although a little smaller in area. Although this study mostly documents the impact of these installations, we did record a limited few instances of no impact, the reason for this requires further study and may help mitigate further loss of seagrass from singing chain moorings. The findings of the present study confirm the need for more widespread use of seagrass friendly mooring systems to reduce seagrass loss and to support recovery (Cullen-Unsworth and Unsworth, 2016).

## Author Contributions

BW and RU contributed to study design, data collection, analysis and write up; LC-U and BJ contributed to write up and analysis.

## Conflict of Interest Statement

The authors declare that the research was conducted in the absence of any commercial or financial relationships that could be construed as a potential conflict of interest.

## Appendix

**APPENDIX 1:** Summary of the available literature examining boat mooring impacts on seagrass.

Name of paper	Author(s) and year	Peer reviewed	Key findings	Seagrass species and location
Effect of boat moorings on seagrass beds near Perth, Western Australia	Walker et al., 1989	Peer reviewed	Seagrass loss studied in three regions, the total seagrass loss due to moorings in these regions is 5.4 ha.	*Posidonia australis* Western Australia
Seagrass loss associated with boat moorings at Rottnest Island, Western Australia	Hastings et al., 1995	Peer reviewed	Eighteen percent of seagrass lost between 1941 and 1992, this is due to moorings.	*Posidonia australis* Western Australia
Monitoring environmental impacts of recreational boat anchoring on eelgrass (*Zostera marina L*.) and benthic invertebrates in the Gulf Islands National Park Reserve of Canada	Leatherbarrow, 2006	Gray	No loss of seagrass due to moorings. Invertebrate diversity and abundance lower in areas with moorings and high levels of recreational boat use.	*Zostera marina* Canada
BACI design reveals the decline of the seagrass *Posidonia oceanica* induced by anchoring	Montefalcone et al., 2008	Peer reviewed	Shoot density and rhizome baring negatively impacted by the mooring system used in Prelo cove.	*Posidonia oceanica* Italy
The impacts of anchoring and mooring in seagrass, Studland Bay, Dorset, United Kingdom	Collins et al., 2010	Peer reviewed	Observed that areas of seagrass are kept clear (∼30 m^2^) due to swinging moorings. Limited quantification.	*Zostera marina* South England (United Kingdom)
Management of the seagrass bed at Porthdinllaen Initial investigation into the use of alternative mooring systems	Egerton, 2011	Gray	Ten meters scar radius around each mooring, leading to 4.5% direct loss of bed.	*Zostera marina* Wales (United Kingdom)
Porthdinllaen seagrass bed, Pen Llyn a’r Sarnau SAC: a survey of moorings in the outer harbor and their impact on the seagrass 2012	Stamp and Morris, 2012	Gray	Density and canopy height 25 and 36% lower between 5 and 10 m away from the mooring. No change in density or canopy height after 10 m.	*Zostera marina* Wales (United Kingdom)
A comparison of the impact of “seagrass-friendly” boat mooring systems on *Posidonia australis*	Demers et al., 2013	Peer reviewed	Swing moorings led to a 9 m radius bar patch in seagrass, screw moorings had little impact. Scar size was 254 m^2^.	*Posidonia australis* Jervis Bay in New South Wales
Estimating losses of *Posidonia australis* due to boat moorings in Lake Macquarie, Port Stephens and Wallis Lake	Glasby and West, 2015	Gray	Amount of damage increases with depth and is typically greater in beds of *P. australis than Z. capricorni*	*Posidonia australis* and *Zostera capricorni* Lake Macquarie, Port Stephens and Wallis Lake
Losses and recovery of organic carbon from a seagrass ecosystem following disturbance	Macreadie et al., 2015	Peer reviewed	Fifty years after disturbance from moorings limited recovery present	*Posidonia australis* New South Wales
Impact of mooring activities on carbon stocks in seagrass meadows	Serrano et al., 2016	Peer reviewed	1.3 and 0.9 ha of seagrass lost at two different sites due to moorings. Organic carbon stores have been compromised due to these moorings. No quantification at each mooring.	*Posidonia* sp. Western Australia
